# The Illinois State Board of Health

**Published:** 1889-05

**Authors:** 


### THE ILLINOIS STATE BOARD OF HEALTH.

The lower house of the State Legislature has apparently developed an amount of opposition to the State Board of Health which seriously threatens the existence of that body. It proposes to make no appropriation for the current expenses of the Board, and to repeal that section (section 11) of the Medical Practice Act which compels itinerant vendors of medicines to procure a license from the State Board of Health.

Such action on the part of the House of Representatives can scarcely be the result of careful consideration of the interests of the State, and is, on sanitary grounds if no other, very much to be regretted. The work of the Board is necessarily of such a nature that personal antagonisms inevitably arise, and there may also be sincere differences of opinion in regard to its methods of work, and in regard to the value to the State of the results which it has accomplished. But with all due consideration it must be admitted that the medical profession is, upon these points, better fitted and more competent to judge than the Legislature is, and the weight of opinion in the medical profession of the State is strongly in favor of the retention of a State Board of Health. Among educated physicians and sanitarians who have given careful attention to the subject, Boards of Health are considered necessities in civilized communities. This expression of opinion is so far entirely independent of the question of continuing the existence of the Illinois State Board of Health as now constituted. We wish not to be misunderstood when we say, in this connection, that we regard the work done by this Board as of great value to the State.

If the Legislature is not satisfied with the work of the Board, which is its servant and creature, it ought to specify to what it objects and give corresponding orders. But in the name of fairplay and straightforward dealing, and in the sanitary interests of the State, let it not resort to the ambiguous plan of making the law a dead letter by refusal to appropriate the money necessary for its execution. Amend it or even repeal it if best or necessary, but do not render it inoperative by stealth.

A discussion of the advisability of the repeal of Section 11 of the Medical Practice Act is intentionally avoided in this connection, but an earnest appeal is made to the Legislature not to allow their disapproval of one or more sections of the act, or of the officers appointed for its execution, to be made the occasion for hasty and ill-considered destruction of an agency which is a sanitary necessity for the protection of the people, but not for the medical profession which needs no legal enactments for its protection, and which should not have any.

